# Nutrition Support Therapy for Hospitalized Children with Malnutrition: A Narrative Review

**DOI:** 10.3390/healthcare13050497

**Published:** 2025-02-25

**Authors:** Sheikha Nasser AlQahtani, Sara AlGubaisi, Faisal Ahmed AlHaffaf, Rabab Jamel Makki, Eman Ali Alohali, Raneem Omran AlMadani, Haifa Mujahed AlSagiheer, Mastourah Mousa Al-Otaibi, Hossam Tawakol Mohammed

**Affiliations:** 1 Department of Dietetics, Prince Sultan Military Medical City, Riyadh 11159, Saudi Arabia; 2 Department of Pediatrics, Prince Sultan Military Medical City, Riyadh 11159, Saudi Arabia; 3 Department of Pharmaceutical Services, Prince Sultan Military Medical City, Riyadh 11159, Saudi Arabia

**Keywords:** nutrition support, guidelines, pediatric, malnutrition, parenteral nutrition, enteral nutrition

## Abstract

Nutrition support is essential to improve clinical outcomes and prevent malnutrition-related complications in hospitalized children. This review aims to explore the latest international guidelines and recommendations for nutrition support therapy over the last decade. Many organizations and pediatric societies emphasize the importance of nutrition support therapy and the critical role of nutrition support teams in assessing and managing malnutrition, particularly after screening patients who are at high risk. Although current recommendations address gaps in clinical practice related to nutrition support, minor differences remain across guidelines due to geographical variations among these societies. A unified approach to implementing nutrition support therapy from admission to discharge, with a clear pathway and the involvement of competent healthcare providers, is needed in all healthcare settings. Furthermore, more in-depth systematic reviews, meta-analyses, and consensus statements that integrate guidelines from all societies are required. Such efforts would better support healthcare providers in aligning clinical practices with the highest standards of care.

## 1. Introduction

Complications related to malnutrition in the pediatric population are a significant health concern, as highlighted by numerous societies and organizations [1,2,3,4]. The prevalence of malnutrition among hospitalized children is higher than previously reported by researchers, which is largely due to inadequate screening and assessment processes in many healthcare settings [5]. Malnutrition, as defined by Mehat and colleagues, refers to an imbalance in the intake and utilization of nutrients, leading to deficiencies, excesses, or imbalances that affect an individual’s health and well-being. This condition encompasses both undernutrition (inadequate intake of nutrients) and overnutrition (excessive intake [1]. Malnutrition in the hospital setting, defined as undernutrition, impacts both clinical and non-clinical outcomes, leading to increased morbidity, mortality, length of hospital stay, and healthcare costs [3,4,5]. It is recommended that all admitted patients undergo malnutrition screening using validated tools (6). Furthermore, international guidelines advocate for the early assessment [6,7] of nutritional status and prompt referral to a nutrition support team to ensure timely intervention [7]. Over the last decade, pathways and guidelines for nutrition support therapy practice have emphasized the importance of enhancing their application across healthcare settings through a multidisciplinary approach [8,9]. Based on the patient’s condition, specialized interventions can be designed to meet nutritional needs when regular oral intake is insufficient. These interventions primarily include two modalities: enteral nutrition (EN) and parenteral nutrition (PN) [10]. Clinical nutrition societies, such as the American Society for Parenteral and Enteral Nutrition (ASPEN), the European Society for Clinical Nutrition and Metabolism (ESPEN), and the European Society for Pediatric Gastroenterology Hepatology and Nutrition (ESPGHAN), regularly update their guidelines related to nutrition support therapy [11,12,13]. Some of these guidelines address gaps in clinical practice, while others do not comprehensively address these gaps. This review explores the latest international guidelines and recommendations regarding nutrition support therapy from the last decade. Through integrating recent evidence and updated protocols, this narrative review aims to support healthcare providers in aligning clinical practices with the highest standards of care.

## 2. Nutrition Support Therapy and Teams

The American Society for Parenteral and Enteral Nutrition (ASPEN) defines nutrition support therapy as the clinical provision of nutrients via enteral nutrition (EN), commonly known as tube feeding, or parenteral nutrition (PN), delivered intravenously when oral intake is insufficient to meet a patient’s nutritional needs [10]. Similarly, the Society of Critical Care Medicine (SCCM), in collaboration with ASPEN, describes nutrition support as the administration of enteral nutrition through an enteral access device and/or PN [14]. Additionally, the ESPEN defines nutrition support as the delivery of nutrition supplementation orally, enterally, or parenterally, either in full or as a partial replacement for inadequate oral intake [15]. For proper implementation of nutrition therapy, many societies recommend establishing a nutrition support team (NST), which is a multidisciplinary group typically composed of dietitians, nurses, pharmacists, and physicians, each playing a distinct role and having distinct responsibilities in delivering comprehensive nutrition support to patients, as outlined in Table 1 [7,8,9,16]. The team’s approach is tailored based on the specific contributions of each member. Additionally, other healthcare professionals, such as physiotherapists, psychotherapists, and social workers, may be integrated into the team as needed to address specific patient requirements [7]. This collaborative approach ensures that the full spectrum of patient care needs is met.

## 3. Nutrition Screening, Referral, and Assessment

Nutrition screening is widely endorsed by major health organizations, such as ESPEN, ASPEN, and ESPGHAN [9,11,17]. These organizations recommend routine nutrition screening for all hospitalized children on admission and during hospitalization, particularly those at higher risk due to chronic illness, feeding difficulties, or socioeconomic challenges [11,17]. Using nutrition screening tools is essential for identifying malnutrition risk or changes in risk, and they should be designed to facilitate a rapid and straightforward screening process [18]. These tools should be usable by non-nutrition-trained staff to ensure broad application in clinical settings. The key parameters that are typically included are dietary intake or appetite, clinical information related to medical conditions affecting nutritional status or treatments impacting nutrition, and one or more anthropometric measurements, including concerns about weight or growth [18]. Tools demonstrating moderate to high validity, reliability, and inter-rater agreement are preferred, such as the Screening Tool for the Assessment of Malnutrition in Paediatrics (STAMP) and the Paediatric Yorkhill Malnutrition Score (PYMS) [19]. The goal is to identify malnutrition risks early, including both undernutrition and overnutrition, with screening integrated into hospital and outpatient settings [19]. ESPEN and ASPEN emphasize the importance of regular monitoring and follow-up to ensure timely interventions and promote healthy growth and development [11,17].

Nutrition referral systems, as outlined by leading organizations, emphasize early identification and referral of children at nutritional risk to specialized teams, particularly in cases of growth failure, severe malnutrition, or chronic medical conditions affecting nutrition [9,11,17]. These referrals are essential for ensuring timely intervention, individualized care, and ongoing nutritional monitoring, often involving multidisciplinary teams.

Nutritional assessment is a structured and comprehensive process designed to identify children with malnutrition or those requiring nutritional support [15]. The assessment includes anthropometric measurements (e.g., weight-for-age, height-for-age, and BMI-for-age) plotted on standardized growth charts from the World Health Organization (WHO) for children under 2 years and from the Centers for Disease Control and Prevention (CDC) for older children to track growth patterns and identify deviations [1,14]. Dietary intake evaluation uses methods such as 24-h recalls and food frequency questionnaires to assess the adequacy of caloric and nutrient intake [18]. Additionally, biochemical markers (e.g., serum proteins and micronutrients) and clinical examinations are employed to detect underlying conditions, such as chronic diseases or malabsorption syndromes, that affect nutritional status [18]. Regular monitoring and reassessment are crucial, especially for high-risk children, to adjust nutritional interventions (e.g., enteral or parenteral nutrition) and ensure proper growth [14,20].

All guidelines stress the importance of a multidisciplinary approach to effectively manage and improve the child’s nutritional outcomes [7,9,11,17].

## 4. Enteral Nutrition

### 4.1. Enteral Nutrition

Enteral nutrition (EN) has been defined as the delivery of nutrients or food into the gastrointestinal tract distal to the esophagus through specifically designed tubes inserted through the nose, mouth, or stoma [21]. EN is recommended for children who are unable to meet their nutritional requirements orally [22]. The timing may vary based on the child’s condition. For instance, in cases of severe illness or metabolic stress, earlier initiation is crucial to meet increased nutritional demands [23]. EN is preferred over parenteral nutrition (PN) in children with a functional gastrointestinal tract (GI), as it is the most physiological form of nutrition, is safer, supports immunity, and is less expensive [24].

#### 4.1.1. Indications and Contraindications

The use of EN should be considered after a number of other oral interventions have been attempted. The first criterion for starting EN is insufficient oral intake, particularly in children who are unable to meet ≥60–80% of individual requirements for ≥5 days in children >1 year of age or for ≥3 days in children <1 year of age. Another criterion is children who meet the criteria for failure to thrive, wasting, and stunting. EN is also appropriate when the total feeding time of a child exceeds 4–6 h per day. Additionally, diet modification can be used as a treatment for conditions such as Crohn’s disease, food intolerance, and metabolic disorders [22,25]. More in-depth pediatric clinical implications for starting EN are mentioned in Table 2.

Furthermore, EN provides a wider range of nutrients, including glutamine, long-chain polyunsaturated fatty acids, short-chain fatty acids, and fiber. In addition to promoting pancreatic and biliary secretions, EN helps promote endocrine, paracrine, and neural factors that support the physiological and immunological integrity of the gastrointestinal tract [26]. It is recommended by the ESPGHAN Committee on Nutrition that EN be used in patients with at least a partially functional gut and an insufficient normal oral intake [13]. Early initiation of EN is associated with improved clinical outcomes, including reduced infection rates, shorter hospital stays, and better recovery from malnutrition [23].

A list of contraindications to start EN is mentioned in Table 3 [22]. As a result of these clinical conditions, EN should be provided to the maximum extent tolerated by the patient along with PN to make up for any nutritional deficiencies. In fact, a small amount of nutrients in the GI can be beneficial [25]. The placement of a percutaneous endoscopic gastrostomy tube (PEG) is contraindicated in patients with obstruction of the pharynx and/or esophagus, malrotation, and interposition of the colon [27].

#### 4.1.2. Administration

- Sites

After determining that EN is necessary, the clinician must determine the most appropriate enteral access method based on each individual’s clinical circumstances. A patient’s GI tract anatomy and function, the indication for feeding, the expected duration of feeding (short-term or long-term), and the likelihood of aspiration all influence the choice of enteral access [28]. There are two main methods of EN delivery: gastric and postpyloric. Gastric feeding is preferred whenever feasible due to its ease of placement, physiological benefits, and ability to accommodate larger feeding volumes through bolus administration [24]. The key differences between bolus and continuous feeding are outlined in Table 4 [24].

When the stomach is functionally and structurally normal, patients usually tolerate gastric feeding. Bolus feeding may result in improved gastric contractility since insulin secretion peaks and troughs may occur with each feeding. Moreover, gastric feedings have a protective effect against hyperosmolar formulas, as duodenal osmoreceptors regulate gastric emptying. This reduces the risk of dumping syndrome, which is a result of inappropriate gut hormone release and causes vasomotor GI symptoms such as abdominal pain, bloating, and diarrhea. However, there is a potential risk of aspiration pneumonia associated with gastric feeding [29]. Therefore, precaution is recommended when providing gastric feedings to patients with severe gastroparesis or a history of aspiration.

Postpyloric feedings are used to overcome difficulties relevant to gastric emptying, gastroesophageal reflux disease (GERD), aspiration, gastroparesis, and gastric outlet obstruction. Children with neurological impairments who are unable to protect their airways may benefit from postpyloric feeding. Bolus feeds should not be delivered postpylorically, as this can result in dumping syndrome [25]. However, continuous infusions provide constant mucosal stimulation, which is imperative for intestinal adaptation and optimal absorption. Additionally, it has a lower risk of emesis and is more effective in achieving enteral balance and weight gain than bolus feeding. As a result, the development of taste or oral motor function may be adversely affected [22]. Conversely, in neurologically impaired children with GERD, King et al. observed more GERD-related and/or dysfunctional swallowing upon switching from gastric to postpyloric feeds [30]. One of the major disadvantages of postpyloric feeding is the difficulty of placement of tubes and the risk that they may migrate back into the stomach. In addition, the tubes are long and of small diameter, which increases the chances of occlusion.

- Routes of enteral access

EN delivery should be determined according to the duration of EN and the underlying disease of the patient or the structure and functioning of the gastrointestinal tract. The feeding tube type should be chosen in accordance with the intended use of the tube, its diameter or French size, and its insertion length for each individual patient [24]. A variety of devices are available that are intended to be used for 12 weeks or fewer, including orogastric, nasogastric, and nasojejunal tubes. This method is the most common and effective form of short-term EN supply; however, the small luminal diameter increases the possibility of blockage. For the purpose of preventing associated sinus and ear diseases, nasogastric tubes should be changed every one to three weeks. Tubes made of polyurethane and silicone are soft and flexible, and they can be left in place for up to six weeks [25]. An abdominal radiograph provides the best method of verifying the tube’s position [31]. The use of nasojejunal tube feeding is an option when gastric feeding is not possible due to the risk of aspiration [22]. Using fluoroscopic guidance, the tip of the tube should be placed distal to the ligament of Treitz [21]. It is recommended that nasogastric or nasojejunal feeding be tried before the placement of a long-term access device, as this will enable the patient to improve their nutritional status, establish a feeding plan, and increase their family’s comfort in taking care of a child with an enteral device.

Long-term enteral nutrition access devices are typically placed when EN is required for more than 3 months. This includes gastrostomy tubes (GT), gastrojejunostomies (GJ), and jejunal tubes (JT). The placement of gastrostomy tubes can be performed endoscopically, surgically, or radiographically [13]. Although endoscopic techniques are the most commonly used method, laparoscopic, fluoroscopic, and surgical techniques are also widely utilized. A percutaneous endoscopic gastrostomy (PEG) tube is placed through transillumination of the abdomen using minimally invasive endoscopy. Following the formation of the tract (6–8 weeks after placement), the PEG tube can be replaced with a more convenient low-profile device. However, the patient needs to undergo a second anesthesia procedure for the change [32]. In recent years, one-step low-profile gastrostomy insertion has become increasingly popular, carrying the same level of complications and outcomes as standard PEG insertion. When a one-step low profile is used for initial placement, T-fastener clips are required for gastropexy. Using T-fastener clips, the stomach wall is secured to the abdominal wall. However, the placement of T-fastener clips can be difficult to use in younger patients [33,34]. It is absolutely contraindicated in those with active sepsis or peritonitis, massive ascites, intractable coagulopathy, and portal hypertension with significant varices. A few relative contraindications include peritoneal dialysis, the presence of ventriculoperitoneal shunts, severe scoliosis, prior abdominal surgery, and morbid obesity [27]. Prophylactic antibiotic administration is recommended to prevent infection at the PEG site and the associated complications [35]. Following the procedure, the patient is allowed to begin a diet six hours after the procedure and to receive full feedings 24 h afterward. There is, however, a higher risk of complications in small children. These include inadvertent bowel injury, gastrostomy leaks that lead to peritonitis requiring laparotomy, and serious hemorrhage [21]. A gastrostomy tube can also be inserted surgically, laparoscopically, or through open gastrostomy. A surgical approach may be preferred in patients suffering from severe scoliosis, those with altered anatomy, or those who require fundoplication simultaneously [27].

In recent years, gastrojejunal and jejunal access devices have become increasingly popular. They are safe and effective methods of enteral feeding in cases in which gastric feeding is inadequate to provide sufficient calories or when there is a high risk of aspiration. They are used primarily in cases of neurological impairments. Ideally, a multidisciplinary team should be involved in the decision to place these tubes and provide active follow-ups and care following their placement [36]. In terms of design, the percutaneous endoscopic gastrojejunostomy tube (PEG-J) is similar to gastrostomy buttons in that the tubes have a low profile, contain a one-way valve to prevent leaks, and are held in place by a retention balloon. There is, however, an additional long distal jejunal tube component that can be customized to fit the needs of individual patients [21]. Until recently, PEG-J tubes were only used by older children and adolescents due to the size limit of 16F, which is not suitable for younger children [37]. A 14F PEG-J button has become available recently and has proven useful in smaller children who require both jejunal access for feeding and gastric venting [38]. Unless an established gastrocutaneous tract exists, a GJ button can be placed using combined laparoscopic and endoscopic methods [25]. It has been shown that direct jejunostomy tubes (PEJs) provide more stable and secure jejunal access for long-term postpyloric feeding compared with PEG-J extensions, with fewer reported complications of blockage or displacement, which consequently reduces radiological and endoscopic intervention [36].

- Feeding Volume According to Bolus and Continuous Feeding

Choosing between bolus feeding and continuous feeding for children is crucial, as the two methods have separate protocols for the volume and timing of feeding. While each method of enteral nutrition has advantages and disadvantages, there is currently insufficient evidence to recommend continuous feeding or bolus feeding. Recently, Theodoridis et al. conducted a systematic review to evaluate the efficacy of continuous feeding compared with bolus feeding [39]. A high risk of bias and heterogeneity in the reporting of clinical outcomes was observed in the included studies, and a conclusion cannot be drawn as to which method is most effective [39]. Nevertheless, bolus feeding may be more appropriate in certain circumstances, such as in infants born with a low birth weight [40]. In practice, a combination of both methods may be employed based on individual patient needs and tolerances, especially in cases where oral skills need to be preserved or when nighttime continuous feeds are beneficial [22].

The initiation and advancement in enteral nutrition should be individualized based on the patient’s clinical condition, stability, and associated risk factors. Medically stable patients can generally achieve target nutritional intake within 1 to 2 days through a more rapid progression [22]. In contrast, medically fragile patients require a gradual approach with close monitoring over the first 3 to 5 days to ensure adequate nutrient delivery and mitigate risks such as refeeding syndrome [22]. Table 5 outlines the initiation process, advancement strategies, and proposed goal volumes for enteral feeding [22,28]. Involving the family in the design and implementation of the EN regimen is crucial, especially for patients who will be on home tube feeding. The family is responsible for administering daily feedings, making it imperative that the feeding schedule accommodates their needs as well.

### 4.2. Energy and Macronutrient Requirements (Protein, CHO, FAT)

According to guidelines from ESPEN, ASPEN, and other pediatric nutrition authorities, energy and macronutrient requirements for pediatric patients are carefully individualized based on the child’s age, growth stage, clinical condition, and metabolic needs [1,18,41].

Energy requirements typically range from 90 to 120 kcal/kg/day for infants and decrease to 50–70 kcal/kg/day for older children and adolescents, though they can increase in cases of illness or metabolic stress [18]. ASPEN and ESPEN recommend the use of indirect calorimetry in pediatric patients, particularly for those who are critically ill or have complex medical needs [1,15]. This is considered the gold standard for measuring energy expenditure and is especially useful when predictive equations are unreliable (18). If indirect calorimetry is unavailable, guidelines suggest using predictive equations (e.g., Schofield, WHO estimates) but emphasize their limitations, particularly in patients with altered metabolism [18]. Usually, the Scofield equation is widely used to estimate the basal metabolic rate (BMR) in children and adults [18]. It is often a starting point for calculating a child’s energy needs, especially in clinical settings. After calculating the BMR, it is typically adjusted by adding an activity factor and/or a stress factor to account for illness or injury [1,41].

Protein needs are crucial for growth and recovery, ranging from 2 to 3 g/kg/day in infants and decreasing to 0.8–2 g/kg/day in older children [1,18]. These needs increase significantly in critically ill or malnourished patients to support tissue repair and immune function [18]. Carbohydrates, the primary source of energy, should provide 45–65% of total caloric intake, with higher carbohydrate needs in infants and younger children [18]. In conditions such as respiratory failure, carbohydrate intake may be restricted to prevent excessive CO_2_ production [18]. Fat requirements are essential for brain development and energy density, contributing 30–50% of total energy intake depending on the child’s age, with younger children and infants requiring higher fat intake [18]. Special attention is given to the inclusion of essential fatty acids to prevent deficiencies, and medium-chain triglycerides (MCTs) are often recommended for children with fat malabsorption issues (e.g., cystic fibrosis) [18].

Regular monitoring and reassessment of nutritional status are critical for adjusting nutrient delivery based on growth, biochemical markers, and clinical progress, ensuring the prevention of malnutrition and optimizing recovery. These guidelines emphasize the importance of early intervention and individualized care to meet the specific needs of pediatric patients, especially those with chronic conditions or critical illness.

### 4.3. Formula Selection

In hospitalized pediatric patients, enteral formula selection depends on age, medical condition, nutritional needs, and tolerance to specific nutrients.

#### 4.3.1. Infant Formulas

Human milk is optimal for most infants and can be fortified with liquid or powdered fortifiers to meet the higher nutritional demands of preterm and term infants [42]. Standard infant formulas, which are generally based on cow milk, are formulated to meet the nutrient requirements of healthy infants up to one year of age, with options such as reduced-lactose or lactose-free formulas that replace lactose with polysaccharides to aid mild lactose sensitivities. However, these are unsuitable for infants with cow milk protein intolerance [18,42]. Iron-fortified soy formulas, which contain higher calcium and phosphorus to compensate for their decreased mineral bioavailability, are suitable for conditions such as galactosemia and hereditary lactase deficiency. Hypoallergenic extensively hydrolyzed protein formulas containing free amino acids and small peptides are appropriate for infants with severe protein intolerance or malabsorption [18,42]. Amino acid-based formulas are indicated for infants who cannot tolerate hydrolyzed formulas, including those with severe allergies, eosinophilic gastrointestinal disorders, or short bowel syndrome [18,42]. Rice starch-thickened formulas are available for reflux reduction by thickening in the stomach, though adding rice or oat cereal directly to formulas disrupts the macronutrient balance [18,42]. For premature infants, specialized preterm formulas that are high in calories, protein, vitamins, and minerals are designed to address nutrient absorption challenges and support growth, compensating for nutritional deficits associated with prematurity [18,42].

#### 4.3.2. Pediatric Formulas

Pediatric enteral product options are expanding, with various formulas being tailored to specific disease states, nutrient needs, and consumer preferences. Most standard pediatric enteral formulas that contain vitamins and minerals meet the micronutrient requirements for children ages 1–10 at an intake of 1000 mL per day [18]. In some cases, adult formulas may be appropriate for children when specific disease states or nutrient needs are identified [18,43]. Pediatric elemental enteral formulas, designed as sole-source nutrition for children with malabsorption issues or multiple food allergies, use 100% free amino acids as the protein source, though the macronutrient content varies across brands [18,43]. Semi-elemental or hydrolyzed formulas are formulated for children with malabsorption or intolerance to polymeric formulas, and they contain peptides derived from hydrolyzed whey or soy protein and typically offer a higher fat content from medium-chain triglycerides (MCTs) than polymeric formulas; they are available in 1 kcal/mL and 1.5 kcal/mL concentrations [18,43]. Pediatric polymeric complete enteral formulas are intended for children with normal gastrointestinal function and are used in tube feeding; these come in flavored and unflavored options, with choices including milk-based, lactose-free, soy, and pea protein versions, available with or without fiber and in caloric densities of 0.67 kcal/mL, 1 kcal/mL, and 1.5 kcal/mL [18,43]. Whole-food-based pediatric formulas vary, with some being formulated for sole-source nutrition and others as supplemental options requiring micronutrient analysis and supplementation. These formulas may use plant- or meat-based proteins and have caloric densities ranging from 1.1 to 1.4 kcal/mL, often requiring dilution for safe and effective administration. Specialized pediatric enteral formulas also include options for ketogenic diets, modified fat content, renal disease, and metabolic conditions [18]. Additionally, modular products are available to enhance the caloric, protein, or fat content of a formula, enabling customized adjustments to meet individual nutritional needs. Table 6 provides an overview of modular sources and their classifications [18]. Selecting the appropriate formula involves careful consideration of each child’s unique nutritional needs, underlying medical conditions, and tolerance for specific formula components.

#### 4.3.3. Metabolic Formulas

Inborn errors of metabolism occur due to enzyme deficiencies in metabolic pathways, leading to substrate accumulation and product deficiencies. These accumulated substrates or their metabolites can be toxic. Management focuses on reducing toxic substrate levels in tissues and plasma by limiting the intake of nutrients that generate toxic byproducts or enhancing their excretion.

- Amino Acid Disorders

Phenylketonuria (PKU) is a metabolic disorder caused by a deficiency of phenylalanine hydroxylase, the enzyme responsible for catalyzing the hydroxylation of phenylalanine to tyrosine. This enzymatic defect leads to the pathological accumulation of phenylalanine and its derivatives, while tyrosine, a key precursor for neurotransmitter biosynthesis and melanin production, becomes insufficient. If untreated, PKU can result in profound neurodevelopmental deficits, impaired somatic growth, eczematous dermatitis, and hypopigmentation of the skin and hair [44,45]. The objective of the phenylalanine-restricted diet is to provide sufficient phenylalanine for growth while maintaining serum phenylalanine levels within a target range of 2–6 mg/dL throughout the lifespan [46,47]. Metabolic formulas composed of synthetic amino acid blends without phenylalanine are essential when dietary restriction leads to inadequate protein intake for growth. These specialized formulas are enriched with tyrosine to compensate for its deficiency in this condition [46]. Nutritional formulas should be customized for each patient, with a range of commercially available options, such as beverages, gels, and ready-to-consume packs, designed to enhance taste and palatability.

Maple syrup urine disease (MSUD) is a disorder of branched-chain amino acid (BCAA) metabolism caused by a deficiency in the activity of the branched-chain α-ketoacid dehydrogenase complex (BCKDC). This enzyme deficiency results in the accumulation of the BCAAs (leucine, isoleucine, and valine) and their corresponding branched-chain α-ketoacids [48]. The goal of nutrition management for MSUD is to maintain near-normal leucine concentrations, prevent isoleucine and valine deficiency, and provide sufficient energy intake to allow for normal growth and development [49]. The metabolic formula consists of amino acid mixtures free from leucine, isoleucine, and valine supplemented with all required essential micronutrients.

Inborn errors of the urea cycle, which are called urea cycle disorders (UCDs), are a group of disorders affecting the enzymes or transporters associated with the urea cycle [49]. Due to the enzymatic deficiency, ammonia may accumulate, resulting in lethargy, coma, or fatal outcomes. The primary objective of medical nutrition therapy for individuals with urea cycle disorders (UCDs) is to mitigate nitrogen load by restricting dietary protein intake, thereby preventing hyperammonemia. The specialized metabolic formula comprises essential amino acid blends fortified with L-cysteine and L-tyrosine and supplemented with essential vitamins, minerals, and trace elements [49].

Methylmalonic acidemia (MMA) and propionic acidemia (PA) are rare, autosomal recessive, multisystemic inborn errors of branched-chain amino acid metabolism that cause significant morbidity and mortality in infancy and childhood [44]. Patients with MMA or PA follow a diet with limited intake of isoleucine, methionine, threonine, valine, and odd-chain fatty acids through regulated protein restriction. Metabolic formulations comprise amino acid blends excluding methionine, valine, threonine, and isoleucine, enriched with essential micronutrients, including vitamins, minerals, and trace elements, to ensure nutritional sufficiency [44].

- Disorders of Carbohydrate Metabolism

Classic galactosemia is an inborn error of galactose metabolism that results from a deficiency of the galactose-1-phosphate (Gal-1-P) uridyltransferase. Early initiation of a lactose-restricted formula or elemental formula may prevent the life-threatening complications of this disorder [18].

Glycogen storage diseases (GSDs) result from a deficiency of enzymes involved in the synthesis or use of glycogen, primarily in hepatic and/or muscle tissue, although other tissues, such as heart or brain tissues, can be involved [50,51]. Glycogen Storage Disease Type 1a (GSD 1a) represents one of the most critical forms of glycogen metabolism disorders. Individuals with GSD 1a are prone to severe hypoglycemia, even following short fasting durations. The primary aim of dietary management is to maintain euglycemia (70–100 mg/dL) while minimizing metabolic disturbances, including dyslipidemia, hyperuricemia, and associated complications such as growth retardation. Limiting fruit (fructose), sugar (sucrose), and dairy products (lactose) is recommended [52,53].

- Fatty Acid Oxidation Disorders

Fatty acid oxidation disorders stem from enzymatic deficiencies that impair the β-oxidation pathway of fatty acids. The primary objectives of nutritional intervention for these conditions are to mitigate fatty acid catabolism by preventing fasting and to ensure sufficient alternative energy substrates, particularly non-lipid sources, during periods of metabolic stress [18].

Medium-chain acyl-CoA dehydrogenase (MCAD) is a crucial enzyme in mitochondrial fatty acid β-oxidation, facilitating hepatic ketogenesis, which serves as a key energy source when liver glycogen reserves are exhausted due to extended fasting or increased metabolic demands [18].

Very-long-chain acyl-CoA dehydrogenase (VLCAD) is critical for the β-oxidation of long-chain fatty acids (14–20 carbons) [18]. Enzyme deficiency leads to the accumulation of these fatty acids and their CoA derivatives, impairing energy metabolism [18]. Nutritional management focuses on reducing reliance on long-chain fatty acids by restricting their intake and preventing fasting to avoid metabolic crises [18]. Specialized MCT-enriched formulas provide an alternative energy source, while breast milk or standard formulas can supply necessary long-chain fats. Essential fatty acids are supplemented through oils such as canola, walnut, or flaxseed oil [53].

### 4.4. Monitoring

Effective monitoring of enteral nutrition in children is essential for promoting healthy growth and development. By employing a combination of clinical assessments, laboratory tests, and regular adjustments to feeding regimens, healthcare providers can optimize nutrition support tailored to each child’s unique needs. Regular monitoring of nutritional status and growth not only helps in addressing immediate nutritional requirements but also plays a crucial role in long-term health outcomes [22]. During regular follow-ups, feeding tubes can be changed every 3 months to a year, depending on the tube.

### 4.5. Complications of Enteral Nutrition

#### 4.5.1. Mechanical Complications

In most cases, these complications are not serious. These include tube migration, clogging, tube breakage, nasolabial irritation, and epistaxis [25]. After gastrostomy or jejunostomy tube insertion, granulation tissue may develop, which can be treated with an application of silver nitrate in moderate to severe cases. A large stoma site may, in some instances, result in leakage of gastric content, causing skin irritation and erythema [27]. A rare but serious complication, buried bumper syndrome, occurs when the internal bumper becomes partially or completely covered with gastric mucosa and migrates out of the stomach as a result of an excessively tight external fixation. In most cases, tubes can be removed and replaced endoscopically [54,55].

#### 4.5.2. Infectious Complications

A variety of complications may occur after tube insertion, such as a wound infection manifested as purulent discharge and cellulitis leading to abscesses. Thus, antibiotic prophylaxis is recommended before tube insertion [35]. A potential late complication may arise due to formula or delivery set contamination and extended hanging times, especially in home-based enteral nutrition [18]. Educating caregivers is crucial to minimizing these risks [22]. Table 7 outlines the recommended hang times for enteral formulas.

#### 4.5.3. Gastrointestinal Complications

There are several symptoms of formula intolerance, including diarrhea, constipation, delayed gastric emptying, and vomiting. To improve symptoms, all possible causes and interventions must be examined [28]. Diarrhea can also be caused by an intolerance to bolus feeds, high infusion rates, high feed osmolarity, and microbial contamination [22].

### 4.6. Refeeding Syndrome

When abrupt feeding of high-energy formula is delivered to patients with malnutrition, particularly with carbohydrates, an endogenous insulin surge will develop. This surge facilitates the shift of electrolytes such as phosphate, potassium, and magnesium from the bloodstream into the cells. As a result, the serum levels of these electrolytes can drop dramatically, leading to heart failure, arrhythmia, and death. Therefore, the initial caloric intake should be <75% of the requirement [56].

### 4.7. Drug–Nutrient Interaction

It is not advisable to dispense coated or slowly degrading medications through an enteral tube. Therefore, using a liquid preparation is the recommended way for medication delivery. However, gelatin capsules should be dissolved in warm water prior to administration, and pills should be blended with water [13].

### 4.8. Transition to Oral Feeding

Weaning children from prolonged EN is a complex process that requires a multidisciplinary approach to ensure a successful transition to oral feeding. Research indicates that various factors, including age, medical condition, and individualized weaning programs, significantly influence the outcomes of this process. A successful weaning program involves collaboration among pediatricians, gastroenterologists, dietitians, psychologists, and speech–language therapists. Programs should be tailored to the child’s specific needs, with a focus on the gradual reduction in tube feeding [28]. Based on a cross-sectional survey conducted by Dipasquale et al., four main eligibility criteria for EN weaning have been identified, namely, stable nutritional status, good oral skills, functional swallowing, and parental motivation [57]. Younger children tend to have better outcomes, and children weaned at a younger age reported lower parental concerns about eating abilities and had better outcomes [58]. Moreover, the literature suggests prolonged EN (over one year) and older age (over 4 to 5 years) are factors contributing to tube weaning failure in young children [57].

## 5. Parenteral Nutrition

### 5.1. Parenteral Nutrition

Oral intake and enteral feeding are always the first choices for delivering nutrients and calories. When the gastrointestinal tract is partially functional, the enteral route should be preferred, even for partial nutrient provision [59]. Parenteral nutrition (PN) is considered non-physiological, as it directly administers nutrients into the bloodstream, bypassing both the digestive tract and portal circulation [59]. In instances where the gastrointestinal tract is inaccessible, all nutrients must be supplied via the parenteral route, a practice referred to as total parenteral nutrition (TPN) [59]. TPN should be employed as a last resort, reserved for situations where oral and enteral feeding are not feasible [59].

#### 5.1.1. Indications and Contraindications

Table 8 shows the common indications for PN [18]. However, the indications are different according to each patient’s characteristics, which means that not all patients with the same condition need to start.

In neonates and premature infants, a careful evaluation of the risks and benefits of PN is crucial, considering factors such as the risk of hypoglycemia versus the potential for infection [60]. PN is typically initiated earlier in premature infants, as they are unlikely to tolerate enteral feeding, with initiation recommended within the first 48 h of life [60]. For infants and children, PN is indicated when enteral feeding is not feasible, and nutritional support is expected to be needed for seven or more days [60]. These patients should undergo evaluation within three days to ensure PN is initiated within four to five days [60]. The timing of PN initiation is guided by patient-specific factors, including age, baseline nutritional status, and underlying medical conditions [61].

PN should be used cautiously in children with metabolic or organ dysfunction, with electrolyte imbalances corrected beforehand [62]. Enteral nutrition remains the preferred method, even in pediatric oncology patients, as concerns exist regarding PN use during chemotherapy [62].

#### 5.1.2. Administration

PN can be delivered through central or peripheral venous access, with the choice largely dependent on the duration of therapy and osmolarity limitations [63]. Peripheral PN is restricted to a maximum osmolarity of 1000 mOsm/L in children and 1100–1200 mOsm/L in infants, making it suitable only for short-term use, typically in conjunction with enteral feeding [63]. Central venous access, which allows for higher osmolarity solutions, is preferred for long-term PN but comes with an increased risk of catheter-related infections and other complications [63].

Several types of catheters are used for PN administration. Non-*tunneled central catheters*, *inserted* into *the subclavian*, *jugular*, *or femoral veins*, *are appropriate for short-term* use (1–2 *weeks*) due to their ease of removal *and* replacement but carry *a* higher risk of *infection* [18,63]. *Tunneled cuffed catheters*, *surgically* placed *in the jugular or cephalic veins*, are preferred *for long-term PN, including home*-based therapy, as they have *a lower infection* risk, though they require surgical *placement and removal* [18,63]. *Peripherally inserted central catheters (PICCs*): These can be placed via any peripheral vein (but are typically placed via the basilic vein), usually inserted via the *antecubital vein*, *are* used *for medium-term PN* and can be managed *in* home settings with proper care [18,63]. Implanted ports, completely subcutaneous devices, are reserved for long-term PN requiring frequent access [18,63].

Despite the advantages of central venous access, catheter-related bloodstream infections (CRBSIs) remain a major concern. To reduce infection risk, many institutions implement ethanol lock therapy; however, this approach is associated with an increased risk of thrombosis and catheter degradation [64]. Alternative antimicrobial agents, such as taurolidine and taurolidine-citrate, are being explored as potential substitutes for ethanol locks, offering improved infection control while maintaining catheter longevity [65].

### 5.2. Energy and Macronutrient Requirements (Protein, CHO, FAT)

The energy requirements for PN are usually slightly less than those for EN due to the absence of digestion-related energy expenditure. However, the actual amount depends on the patient’s condition, clinical goals, and risk of complications such as overfeeding [1,18,41].

Amino acids are crucial for growth and are delivered through specialized pediatric formulations [66]. For neonates, amino acids must be carefully dosed to prevent protein catabolism while supporting growth [66]. Term infants generally receive 2.5–3 g/kg/day, while preterm infants might require 3–4 g/kg/day due to higher protein demands [66]. The formulations ensure appropriate nitrogen balance without overburdening immature liver and renal systems, which are less efficient in metabolizing proteins [66]. To prevent additional losses, protein is dosed as high as 4 g/kg/d in preterm neonates receiving “starter PN”. For term neonates, dosing begins at 2–3 g/kg/d with a goal dose of 2.5–3 g/kg/d [18]. Although some practitioners may advance protein provision more slowly (0.5 g/kg/d), there is no evidence to support that practice [18]. Protein doses of 1 g/kg/d have been shown to prevent negative nitrogen balance and minimize the risk of hyperkalemia. To prevent catabolism and additional protein losses, it is important that protein be provided in addition to dextrose soon after birth [18]. Infants who are only provided with dextrose-containing fluids would continue to break down muscle mass, which is difficult to regain [18].

Carbohydrates are administered as dextrose, contributing to caloric intake and preventing hypoglycemia [14]. For neonates, the initial infusion rate is 5–8 mg/kg/min, and this is adjusted based on tolerance and blood glucose monitoring [14]. The goal is to meet energy needs without causing hyperglycemia, which can strain metabolic systems [14]. Carbohydrates typically account for 60–70% of non-protein calories, with gradual increases to match energy requirements [14]. Depending on tolerance, dextrose is gradually advanced to a GIR of 12–15 mg/kg/min. The use of insulin is avoided, and ILE provision can be altered to offset the loss of carbohydrate calories in glucose-intolerant neonates [14].

Lipids provide essential fatty acids (EFAs) and energy, which are crucial for the development of the brain and eyes in neonates [67]. Traditional ILEs based on soybean oil supply omega-6 fatty acids, which can promote inflammation and hepatic strain if used in the long term [67]. Modern emulsions enriched with fish oil or a mixture of oils are preferred to reduce hepatotoxicity and improve tolerability in neonates [67]. The standard starting dose is 1–2 g/kg/day, with adjustments based on serum triglyceride levels, and they are maintained under 250 mg/dL to avoid hypertriglyceridemia [67]. Monitoring serum triglyceride (TG) levels is recommended to assess ILE tolerance, with the target level being below 250 mg/dL. For infants at risk of hypertriglyceridemia, infusions should ideally be administered over 24 h with an infusion rate that does not exceed 0.15–0.17 g/kg/h, depending on the specific ILE [67]. Due to reduced levels of lipoprotein lipase in infant capillaries, which aids in TG breakdown, adding heparin to PN (at 0.5–1 U/mL) can stimulate the enzyme and improve TG clearance. Additionally, levocarnitine, a lysine derivative, can aid in managing hypertriglyceridemia by transporting long-chain triglycerides across cell membranes to the mitochondria [67].

Table 9 presents ASPEN’s recommendations for initiating and advancing parenteral nutrition (PN) in pediatric patients, highlighting the importance of gradual adjustments in protein, carbohydrate, and lipid intake based on metabolic tolerance [18]. Likewise, Table 10 provides guidelines for neonates, emphasizing early amino acid provision, controlled glucose infusion, and progressive lipid administration to support optimal growth and development [18].

### 5.3. Micronutrient Requirements

#### Micronutrient Requirements

Pediatrics require specific electrolyte adjustments due to their rapidly developing systems and high growth demands. Sodium, potassium, calcium, and phosphorus are particularly important [18,68]. Table 11 presents the recommended daily dosage of individual electrolytes for pediatric patients, ensuring appropriate balance to support metabolic functions and overall health [69]. Abnormal fluid losses as the result of diarrhea, vomiting, a high nasogastric tube, or ostomy output can impact serum electrolytes. The estimated electrolyte concentrations for different GI body fluids may be helpful in determining electrolyte replacement dosing [18,68].

1. Gastric fluid has variable sodium and chloride levels (20–80 mmol/L), with minimal bicarbonate, reflecting its acidic nature [18,68].
2. Small intestine fluid contains high sodium (up to 140 mmol/L) and bicarbonate (40 mmol/L) to aid in nutrient absorption and maintain an alkaline environment [18,68].
3. Diarrheal stool electrolyte loss, especially that of sodium (90 mmol/L) and potassium (80 mmol/L) can lead to dehydration and electrolyte imbalances [18,68].
4. Ileostomy output has a broad range of sodium and chloride from 45 to 135 mmol/L, highlighting significant electrolyte loss that requires monitoring [18,68].
5. Colon fluid contains moderate sodium and chloride (60 mmol/L) but minimal potassium and bicarbonate, as water absorption concentrates the contents [18,68].

Sodium is typically administered at 2–5 mEq/kg/day and adjusted for hydration and serum sodium levels [69]. Sodium, as the main ECF cation, impacts intravascular and interstitial fluid volumes and is primarily excreted in urine, with minor losses through sweat and feces [69]. Chloride, the major ECF anion, generally parallels sodium in maintaining extracellular volume balance but can act independently, influenced by bicarbonate equilibrium [69].

Potassium is given at 1–3 mEq/kg/day to support cellular function, with careful monitoring to avoid hyperkalemia [69].

Calcium and phosphorus are delivered in a ratio conducive to bone mineralization, generally 1.7:1 (mg), and they are crucial for preterm infants prone to metabolic bone disease due to rapid bone development. Both calcium and phosphorus are important for the maintenance of electric impulses [69].

Magnesium is important for coenzyme activation and calcium and potassium homeostasis [69].

### 5.4. Fluid and Electrolyte Requirements

Neonates and infants have higher fluid requirements due to their immature kidneys and greater insensible water losses, particularly through the skin [68,69]. Standard fluid volumes range from 80 to 150 mL/kg/day for preterm infants, and they are modified for factors such as fever or renal function [68,69]. The Holliday–Segar method is a standard formula for older children, but neonates often need individualized adjustments based on their specific conditions and daily measurements, including their weight, serum sodium, and urine output [70].

#### 5.4.1. General Fluid Requirements by Age and Weight

Fluid requirements in pediatric PN are typically calculated using guidelines that consider age and weight, while those of neonates and infants are generally higher due to their immature kidneys and higher metabolic rates [69]. Preterm neonates start with approximately 40–180 mL/kg/day, and this is gradually increased based on age and clinical stability [69]. For infants, the recommended requirements are often 120–150 mL/kg/day to support growth and development, while for older children, the fluid requirements per kilogram decrease due to children’s growth [69]. In addition, toddlers’ fluid requirements are around 100–120 mL/kg/day [69]. For children (4–10 years), the requirement is roughly 80–100 mL/kg/day, and for adolescents (11+ years), the fluid requirements are calculated using the Holliday–Segar method, which is common in pediatric settings.

For the first 10 kg: 100 mL/kg;

For the next 10 kg: 50 mL/kg;

For each additional kg over 20 kg: 20 mL/kg [69].

These values provide a baseline and should be adjusted based on the child’s hydration status, growth, and illness-related factors.

#### 5.4.2. Adjustments Based on Clinical Conditions

Certain health conditions require modifications to fluid calculations:

1. Critically ill children (e.g., in the ICU) may need fluid restrictions to avoid complications such as edema, respiratory distress, or heart failure. In these cases, the fluid requirements might be limited to the minimum necessary to maintain hydration and electrolyte balance [18].
2. Children with kidney disease often require reduced fluid intake to prevent fluid overload, as their ability to excrete excess fluid may be compromised [18].
3. Burns or fever: These conditions increase insensible water loss, which may necessitate higher fluid intake to replace losses [18].
4. Premature infants have higher fluid needs due to their immature skin and kidneys, which increase water loss. They may require more frequent adjustments based on daily weight changes and lab values [18].

### 5.5. Monitoring and Adjustment

Monitoring is essential to ensure that nutrient levels are adequate and prevent complications [18]. Commonly monitored parameters include blood glucose, serum electrolytes, and triglycerides [18]. In neonates, glucose monitoring is particularly crucial due to their susceptibility to both hypoglycemia and hyperglycemia [18]. Adjustments are made based on daily lab values, with a particular focus on balancing dextrose to prevent metabolic stress [18]. Neonates are susceptible to acid–base imbalances, with metabolic acidosis being a common concern due to renal immaturity [18]. Blood gas analysis helps monitor pH and bicarbonate levels [18]. Chloride-free solutions may be used to prevent metabolic acidosis, and bicarbonate precursors such as acetate can be added to help buffer the blood. Frequent weight monitoring is necessary, especially for neonates [18]. Weight changes provide insights into hydration status and fluid retention [18]. If the infant experiences rapid weight gain or loss, this can indicate fluid imbalances requiring intervention [18]. Electrolytes are also monitored, with specific attention to maintaining optimal levels of sodium, potassium, calcium, and phosphorus to support cellular and bone health [18].

### 5.6. Complications

#### 5.6.1. Parenteral-Nutrition-Associated Liver Disease

Parenteral-nutrition-associated liver disease (PNALD) is when the long-term use of PN, especially with soybean-oil-based ILEs, increases the risk of PNALD, a condition marked by liver inflammation, fibrosis, and even cholestasis [18]. Using omega-3-enriched ILEs, such as those containing fish oil, has been shown to mitigate these risks by reducing liver stress and inflammation [18]. Cycling PN, where infusions are intermittently stopped, also allows the liver to rest [18]. Early enteral feeding is encouraged to stimulate bile flow and gut motility, which can further reduce PNALD risk [18].

#### 5.6.2. Electrolyte Imbalances

- Hypernatremia (Na > 145 mmol/L)

This is primarily iatrogenic in neonates, especially very-low-birth-weight infants (VLBWIs), due to factors such as improper replacement of transepidermal water loss (TEWL), inadequate water intake, or excessive sodium intake. The initial postnatal period is particularly sensitive to these imbalances [69]. Assessment of the infant’s hydration status and intravascular volume is essential [69]. Hypernatremia due to dehydration presents with hypovolemia symptoms [69]. Therapy should correct the cause; symptomatic hypovolemia requires plasma volume replacement [69]. Rapid correction of hypernatremia is avoided to prevent cerebral edema and seizures. The recommended correction rates are 10–15 mmol/L per day to safely reduce sodium levels [69].

- Hyponatremia (Na < 135 mmol/L)

This reflects either water overload or sodium depletion [69]. Water overload might suggest renal issues such as acute renal failure, especially in cases of low urine output and elevated urinary sodium (>20 mmol/L) [69]. Sodium depletion may result from high renal losses due to immature kidney function, which is common in preterm infants born before 34 weeks [69]. Clinical and extracellular fluid (ECF) assessments, along with urinary sodium measurements, help determine the underlying cause. Water overload is seen with insufficient weight loss, while sodium depletion often accompanies ECF contraction and proper weight loss [69], depending on the underlying etiology [69]. Rapid correction over 48–72 h is avoided to prevent risks such as pontine myelinolysis [69].

- Hyperkalemia (K > 6 mmol/L)

Hyperkalemia can occur without renal impairment; it is commonly due to non-oliguric hyperkalemia (NOHK) in VLBWIs, and it is influenced by factors such as acidosis, birth asphyxia, and hemolysis [69]. In oliguric hyperkalemia, typically due to renal failure, potassium excretion is reduced [69]. NOHK shows normal diuresis and elevated urinary potassium (>20 mmol/L), while oliguric hyperkalemia has low urinary potassium (<20 mmol/L) [69]. Severe hyperkalemia (>7 mmol/L) requires urgent treatment, including the use of ion-exchange resins, diuretics, or insulin–glucose infusions to reduce potassium levels [69].

- Hypokalemia (K < 3.5 mmol/L)

This results from increased demand, insufficient intake, or high renal losses, commonly from medications such as diuretics [69]. Early PN stimulates insulin production, promoting potassium transfer into cells, which can lead to hypokalemia if potassium intake is not sufficient [69]. Assessing potassium supply, renal function, and medication effects is essential, especially in growth-restricted neonates or those on early high-protein PN. Potassium intake should be adjusted alongside amino acid supply to avoid hypokalemia and prevent refeeding syndrome, where rapid nutrient provision induces a shift of potassium and phosphate into cells.

- Severe Metabolic Acidosis (pH < 7.2)

This is primarily due to high cumulative chloride intake in PN, which can lead to metabolic acidosis, particularly in infants with large patent ductus arteriosus (PDA) or those experiencing rapid weight loss (>15%) [69]. Excess chloride intake affects acid–base balance, reducing the strong ion difference (SID) and leading to acidosis [69]. Blood gas analysis and monitoring of chloride intake help diagnose metabolic acidosis [69]. High chloride intake (>10 mmol/kg in the first 3 days or >45 mmol/kg in 10 days) is a primary indicator [69]. Using “chloride-free” sodium and potassium solutions in PN can help mitigate the risk of hyperchloremic acidosis [69]. Close monitoring of the acid–base balance is also recommended to adjust PN composition as needed [69].

#### 5.6.3. Infections

Catheter-related bloodstream infections (CRBSIs) are a major concern in pediatric PN, particularly for long-term cases [18]. Preventative strategies include strict aseptic techniques for catheter insertion and maintenance [18]. Any sign of infection necessitates prompt antibiotic treatment, and severe cases may require catheter removal and replacement [18].

### 5.7. Parenteral Nutrition Safety

#### 5.7.1. Safety of the PN Process

The initial step in managing parenteral nutrition (PN) involves a comprehensive assessment of the patient’s nutritional status, followed by the creation of an individualized nutrition care plan [71]. Depending on the healthcare setting, this assessment may be conducted by various professionals, such as dietitians, physicians, pharmacists, or other trained members of the nutrition support team [71]. Once the plan is developed, it undergoes a meticulous review by pharmacists or compounding center staff to confirm the correct dosing, compatibility, and stability [71]. This review is crucial due to the complexity of PN, which can involve 40–50 components and presents a high risk for errors that could lead to severe complications [71].

To minimize errors and standardize PN delivery, organizations such as the Institute for Safe Medication Practices (ISMP) and ASPEN recommend using standardized formulations and order templates [71]. For instance, ASPEN provides a template in its safety guidelines to help ensure accuracy and reduce risks associated with PN [72]. Following verification, the PN prescription moves to the compounding phase, where the pharmacist prepares, labels, and dispenses the mixture under stringent sterile conditions to prevent contamination or dosing errors [72]. In hospitals, nurses typically handle PN administration, while patients or caregivers manage this at home [72]. This involves verifying the PN order, selecting appropriate venous access, and adhering to infection control protocols. Continuous patient monitoring is essential to assess nutritional outcomes and detect complications, such as mechanical or metabolic issues [72]. Throughout the PN process, standardized procedures are vital for minimizing errors, as underscored by guidelines from healthcare safety organizations [72].

According to recent studies, the PN management process highlights the potential errors and error rates at each stage [73]. It emphasizes the roles of healthcare providers and caregivers in ensuring safety and quality across the PN lifecycle [73]. Errors are common care transitions during handoffs between care settings, such as missing information or miscommunication [73]. The error rate may reach 46%, which is the highest rate in the PN process [73]. The second process containing errors is preparation, compounding, and dispensing, where the risks include product mix-up, lack of sterility, missing additives or incompatibilities, and incorrect labeling or storage [73]. The error rate for this process is around 24–42%. Common errors in the administration process include the nurse/patient/caregiver using the wrong patient, dose, drug, route, or time [73], as well as the contamination of the infusion system. The error rate in this process is up to 28–35% [73]. The next process in which errors occur is the order review and verification by a pharmacist/technician [73]. Potential issues in this process include misinterpretations, insufficient evaluation for compatibility or stability, and missing or incorrect ingredients [73]. The error rate in this process is 20–39%. The lowest error rate in this cycle is in prescriptions [73]. Errors may include doses outside the normal range, incorrect volume or infusion rate, and incompatibilities [73]. Their error rate can be up to 1–8%. Regarding the overall error rate, the entire process experiences 3–16 errors per 1000 scripts, highlighting the complexity and high-risk nature of PN management [73].

#### 5.7.2. International Considerations Regarding PN Safety and Quality

All-in-one (AIO) PN admixtures contain around 40–50 components, comprising key macronutrients and micronutrients essential for patient health [73]. These include amino acids (contributing 15–20% of total energy intake), glucose or dextrose (about 50% of energy), and lipid emulsions (providing 30–35% of energy), alongside both water- and fat-soluble vitamins, trace elements, fluids, and electrolytes, all of which are customized to meet individual patient needs [73]. Guidelines, such as those from ESPEN, provide recommendations for stable patients on PN, typically suggesting 20–35 kcal per kilogram of body weight daily and 0.8–1.4 g of protein per kilogram for most long-term, non-critically ill patients [73].

Nutritional requirements may vary based on specific clinical scenarios, such as intensive care, fluid restrictions, chronic kidney disease, or additional energy needs from co-medications [73]. International guidelines from ESPEN and ASPEN offer tailored PN formulations for diverse patient populations, including pediatric and ICU patients [73].

## 6. Conclusions

Nutrition support therapy, which is led by a nutrition support team, plays a vital role in patient care. A multidisciplinary approach is recommended across all healthcare settings in order to ensure the effectiveness and efficiency of nutritional interventions. While oral supplementation is always the preferred method, artificial nutrition support is recommended when oral intake is not feasible. Key gaps in clinical practice related to nutrition support include limited resources, unclear roles and responsibilities within the nutrition support team, inadequate discharge education for patients and caregivers, inconsistent malnutrition screening, poor communication among multidisciplinary teams, insufficient training on updated guidelines, and inadequate monitoring and documentation of interventions. Addressing these gaps requires the implementation of clear policies, the optimization of resources, and enhanced staff training to align with best practices and improve patient outcomes.

## Figures and Tables

**Table 1:** Roles and responsibilities of each nutrition support team member [7,8,16].

Team Member	Role
Dietitians	Provide expert guidance on the use of enteral and parenteral nutrition, including indications, solution selection, and setting nutritional goals.
	Recommend appropriate nutrition options, including specialized nutrition, such as immuno-nutrition, vitamins, and trace elements.
	Develop, implement, and regularly update protocols for enteral and parenteral nutrition, as well as for managing complex nutritional therapies.
	Design and interpret screening tools for assessing nutritional needs and initiate comprehensive nutritional assessments.
	Play a key role in education and actively participate in research on advanced nutritional interventions.
Nurses	Provide guidance on the appropriate routes, methods, and systems for delivering enteral and parenteral nutrition.
	Evaluate and ensure the adequacy of access to nutrition therapy.
	Offer expertise in the use of feeding tubes, pumps, and associated enteral/parenteral equipment.
	Implement and update protocols for delivering enteral and parenteral nutrition to promote standardization, reduce costs, and minimize mechanical complications.
	Educate healthcare staff on enteral/parenteral nutrition practices and the management of complex nutritional therapies.
	Engage in research related to the administration and improvement of complex nutritional therapies.
Pharmacists	Provide logistical support for the preparation and administration of parenteral nutrition.
	Monitor and advise on potential chemical and pharmaceutical interactions between components of parenteral nutrition.
	Offer expertise on the composition, stability, and compatibility of parenteral nutrition formulations, as well as drug interactions with enteral and parenteral nutrition.
	Contribute to the development and implementation of parenteral nutrition protocols.
	Actively participate in and support research related to complex nutritional therapies.
Physicians (usually surgeons, intensivists, or gastroenterologists)	Oversee and direct the administration of enteral and parenteral nutrition therapies.
	Champion the use of nutrition therapy within their specialty area.
	Offer expert guidance for managing complex cases requiring specialized nutritional interventions.
	Engage in and promote research initiatives related to advanced nutrition support.
	Educate and inform peers, trainees, and leadership about the critical role of nutrition therapy in patient care.

**Table 2:** Clinical implications for pediatric patients utilizing enteral nutrition [22,25].

Insufficient oral intake	Abnormal sucking and swallowing Congenital anomalies in the upper gastrointestinal tract Severe gastroesophageal reflux Critical illness Food aversion, anorexia nervosa, and depression
Disorders of digestion and absorption	Short bowel syndrome Congenital anomalies in the gastrointestinal tract Gastrointestinal dysmotility Intractable diarrhea in infancy Cystic fibrosis Autoimmune enteropathy and immunodeficiency Pancreatitis Graft versus host disease
Increased nutritional demand	Inflammatory bowel disease Cystic fibrosis Extensive burn Chronic solid organ disease: heart, liver, kidney
Conditions for which enteral nutrition is essential to the management of the illness	Crohn’s disease Inborn error of metabolism Ketogenic diet in epilepsy

**Table 3:** A list of contraindications to start EN [22].

Absolute contraindication	Gastrointestinal dysfunction: Paralytic ileus Mechanical ileus Intestinal obstruction Intestinal perforation
Relative contraindications	Intestinal dysmotility Necrotizing enterocolitis Toxic megacolon Diffuse peritonitis Gastrointestinal bleeding Enteric fistulas with high output

**Table 4:** Key differences between bolus and continuous feeding [24].

Feature	Bolus Feeding	Continuous Feeding
Volume per feed	Larger volumes at intervals	Smaller volumes continuously
Frequency	Every few hours	Continuous over many hours
Physiological mimic	More natural eating pattern	Less mimicking of natural eating
Risk of aspiration	Higher risk due to larger feeds	Lower risk with steady delivery
Nutritional absorption	Cyclical hormone surges improve absorption	Steady hormone levels

**Table 5:** Initiation, advancement, and proposed goal volumes of enteral feeding [22,28].

	Bolus Feeding			Continuous Feeding		
	0–12 Months	1–6 Years	>7 Years	0–12 Months	1–6 Years	>7 Years
Initiation	10–15 mL/kg every 2–3 h	5–10 mL/kg every 2–3 h	90–120 mL/kg every 3–4 h	1–2 mL/kg every hour	1 mL/kg every hour	25 mL/kg every hour
Advancement	10–30 mL per feeding	30–45 mL per feeding	60–90 mL per feeding	1–2 mL/kg every 2–8 h	1 mL/kg every 2–8 h	25 mL every 2–8 h
Goal volume	20–30 mL/kg every 4–5 h	15–20 mL/kg every 4–5 h	330–480 mL every 4–5 h	6 mL/kg every hour	1–5 mL/kg every hour	100–150 mL every hour

**Table 6:** Source of modulars and classifications [18].

Source of Modulars	Classifications
Protein	Liquid or powder Intact or extensively hydrolyzed Pureed baby food meats May alter the osmolarity of the formula significantly
Carbohydrate	Liquid or powder Table sugar, juice, corn starch May significantly alter the osmolarity of the formula
Fat	MCTs or LCTs Combination products with varying contents of carbohydrate, protein, or fat available. Formula safe

**Table 7:** Recommended hang times for enteral formulas [18].

Enteral Formulas	Hang Times
Formula from powder, formula with additives, and human milk	4 h
Sterile, ready-to-feed formula	8 h
Closed system	24 h
Home-blended	2 h

**Table 8:** Indications for parenteral nutrition in newborns, infants, and children [18].

Age Group	Indications
Newborns	<32 weeks of gestation and/or <1500 g >32 weeks of gestation with IF Bridging parenteral nutrition
Infants and Children	Cannot meet nutritional requirements via enteral feeding Burn Multiorgan failure Clinical conditions leading to contraindication to enteral nutrition (paralytic or mechanical ileus, anatomical disruption of the gastrointestinal (GI) tract, intestinal obstruction, GI ischemia, diffuse peritonitis, bowel perforation) Malabsorption: short bowel syndrome, chronic diarrhea, malabsorptive syndrome example (tufting enteropathy, microvillous inclusion disease)

**Table 9:** ASPEN recommendations for the initiation and advancement in parenteral nutrition for macronutrients in pediatrics [18].

	Initiation		Advanced by		Goal	
Macronutrient	Preterm	Term	Preterm	Term	Preterm	Term
Protein (g/kg/day)	1–3	2.5–3	-	-	3–4	2.5–3
Dextrose (mg/kg/min)	6–8	6–8	1–2	1–2	10–14 (max 18)	10–14 (max 18)
Intravenous lipid emulsion (ILE) (g/kg/day)	0.5–1	0.5–1	0.5–1	0.5–1	3 (max 0.15 g/kg/h)	2.5–3 (max 0.15 g/kg/h)

**Table 10:** ASPEN recommendations for the initiation and advancement in parenteral nutrition for macronutrients in neonates [18].

Parameter	Initiation (Children 1–10 Years)	Advance By (Children 1–10 Years)	Goal (Children 1–10 Years)	Initiation (Adolescents)	Advance By (Adolescents)	Goal (Adolescents)
Protein (g/kg/day)	1.5–2.5	—	1.5–2.5	0.8–2	—	0.8–2
Dextrose (mg/kg/min)	3–6	1–2	8–10	2.5–3	1–2	5–6
ILE (g/kg/day)	1–2	0.5–1	2–2.5	1	—	1–2

**Table 11:** Daily dose of individual electrolytes in pediatrics [70].

Electrolyte	Pediatric Dosing	Adolescents/Children Weighing > 50 kg
Sodium	2–5 mmol/kg	1–2 mmol/kg
Potassium	2–4 mmol/kg	1–2 mmol/kg
Chloride and Acetate	As needed to maintain acid/base balance	As needed to maintain acid/base balance
Calcium	0.25–2 mmol/kg	5–10 mmol
Phosphorus	0.5–2 mmol/kg	10–40 mmol
Magnesium	0.15–0.25 mmol/kg/day	5–15 mmol/day

## Data Availability

No new data were created or analyzed in this study.
